# Recent Advances on Diatom-Based Biosensors

**DOI:** 10.3390/s19235208

**Published:** 2019-11-28

**Authors:** Ilaria Rea, Luca De Stefano

**Affiliations:** Institute for Microelectronics and Microsystems, National Research Council, Via P. Castellino 111, 80131 Napoli, Italy; ilaria.rea@cnr.it

**Keywords:** diatoms, biosensors, nanotechnology, porous materials

## Abstract

Porous materials showing some useful transducing features, i.e., any changes in their physical or chemical properties as a consequence of molecular interaction, are very attractive in the realization of sensors and biosensors. Diatom frustules have been gaining support for biosensors since they are made of nanostructured amorphous silica, but do not require any nano-fabrication step; their surface can be easily functionalized and customized for specific application; diatom frustules are photoluminescent, and they can be found in almost every pond of water on the Earth, thus assuring large and low-cost availability. In this review, the most recent advances in diatom-based biosensors are reported, and a perspective view on future developments is given.

## 1. Introduction

**Figure 1:**
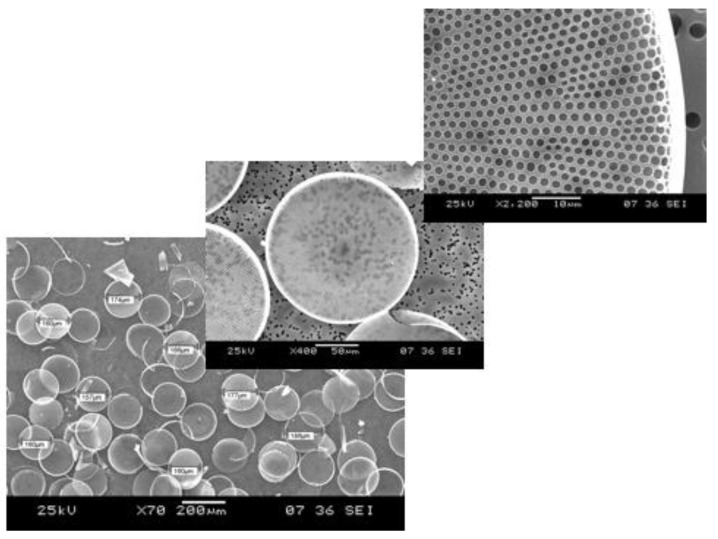
Scanning electron microscope images of the *Coscinodiscus wailesii* diatom valves at different magnifications (Images courtesy of Prof. M. De Stefano, University of Campania “Vanvitelli”, Italy).

Sometimes the term biosensor is improperly used to describe a molecular system that detects a physical or chemical interaction between a probe and its target analyte, such as the antibody (the probe) antigen (the target analyte) couple. A biosensor is instead a working device that must give a numerical value when used to selectively quantify the amount of the target analyte in a complex mixture, just like any other commercial sensor used in the industry or everyday life [1]. A biosensor is made of a transducer element, the true sensitive part of the device, and a receptor that interacts with the target analyte. The advent of nanostructured materials as high-quality transducer supports has strongly improved the performance of biosensors, thus increasing the interest of the academic and industrial world. Among several nanostructured materials engineered in the research laboratories, the porous ones have been deeply investigated since they offered a specific feature with respect to their bulk or planar counterparts and, that is, a huge specific surface, available to sense the molecular probe-target interaction. This is the case, for example, of the porous silicon which is the nanostructured analog of crystalline silicon [2,3]. The porous silicon not only has a sponge-like morphology with a specific area that can be of hundreds of m^2^/g but it could also be photoluminescent. Porous silicon changes its physical properties on exposure to gases or liquids, whereas the crystalline silicon is almost inert and it cannot emit light [4]. Even if the nanostructured porous transducers show superior performances in sensing applications, they require specialized fabrication processes that could be expensive and complex. These are the main reasons why researchers considered some naturally-derived or biomimetic alternatives to man-made materials. Diatoms are microalgae diffused in marine and freshwater all over the planet, largely studied in biology concerning oxygen production and, also, as markers of environmental pollution [5]. Diatoms are constituted by a single cell enclosed in a nanostructured skeleton, called frustule, made of hydrogenated amorphous silica, which has dimensions ranging from few microns up to a millimeter. The frustule consists of two valves joined by girdle bands. The valves are pierced from side to side, as it shown in Figure 1 where images of the diatom *Coscinodiscus wailesii* by scanning electron microscope are reported. A very detailed description on diatom structure can be found in a paper of Losic et al. [6], where scanning electron microscopy and atomic force microscopy results are presented. From the biologic point of view, the frustule provides mechanical protection of the living cell, molecular sieving of nutrients, and light managing.

**Figure 2:**
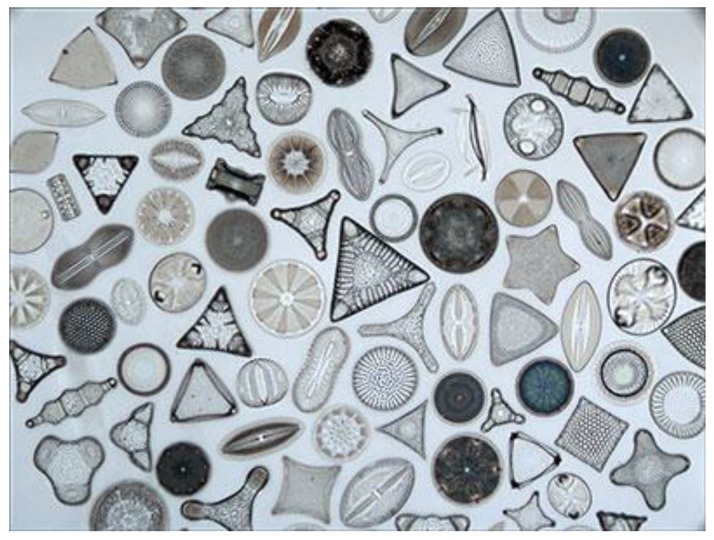
Different shapes, sizes, and morphologies of diatom frustules in an optical image (125× magnification). Frustule dimensions could range from few microns up to a millimeter (Copyright to http://golubcollection.berkeley.edu/diatoms/modern.html).

Frustules show a hierarchical distribution of pores from tenths to hundreds of nanometers in size [7]. Frustules size, pore arrangement, and dimensions are species-specific as can be seen in Figure 2. From the material point of view, once the organic part is removed with acid or basic wet treatments, the diatom frustules are very similar to man-made porous silica, except for the fact that they can be found in nature and do not require any complex fabrication process. Moreover, just like the porous silicon, the diatom frustules have remarkable optical properties that change on exposure to chemical substances [8,9,10,11,12,13,14,15,16]; their surface can be functionalized [17], and in conclusion, the diatom frustules have been considered as bio-derived transducers for biosensing applications [18]. In the last years, due to their unique characteristics, an increasing enthusiasm about the use of diatoms in nanotechnology has been registered in many important fields, from nanomedicine to environment monitoring [19,20,21,22,23,24,25,26,27,28,29,30,31]. Two books have been recently published in the area of diatoms, fixing the state of art in emerging applications [32,33]. Moreover, a recent review summarized the results on the use of diatoms in biosensing until 2015 [34]. In this paper, the new significant papers published in peer-reviewed scientific journals in the last four years will be commented, and some future perspectives about the evolution of diatom-based biosensors will be given.

## 2. Diatom Surface Functionalization

The diatom frustules, i.e., the microshells containing the cells, are mainly made of amorphous silica with a high level of the hydroxyl group (OH) on the surface since the diatoms self-assemble the frustules in a water environment and always stay there. The chemistry of silica functionalization is well known, and diatom frustules can be chemically modified just like the standard glass slide use in immunoassay. The most used route to surface silanol groups (Si-OH) substitution is the silanization, which is the covering of the glassy surface with organofunctional alkoxysilane molecules, such as 3-amino-propyl-triethoxysilane (APTES) or 3-amino-propyl-dimethyl-ethoxysilane (APDMES) [35]. The biogenic cell wall of diatoms can be thus transformed in amine-terminated surface (Si-C-Si-….-NH_2_) that can be used to covalently bind the molecular probes (antibodies, enzymes, proteins, DNA strands, and so on). Selvaraj et al. used some amine-passivated frustules of the diatom *Nitzschia* sp. to detect nitroaromatic compounds with high sensitivity and specificity [36]. Beyond silanization, diatom frustules can be modified by metals deposition or polymers infiltration, depending on the specific design application that could be thought for this particular nanomaterial [37]. Diatoms are usually handled in aqueous solutions, but frustules can be both suspended in a colloidal-like solution and deposited on dry, inert support. For example, Leonardo et al. recently reported how antibody functionalized diatoms have been fixed on metal electrodes via gold electrodeposition [38]. By changing the parameters of electrodeposition (applied potential, time, and gold concentration) it was possible to control the diatom immobilization orientation and yield. This method worked with frustules of different sizes and shapes, resulting in nanostructured electrodes with enhanced performances in electrochemical biosensing. In another very recent proof of concept experiment, the diatom *Phaeodactylum tricornutum* surface has been modified by ZrO_2_ by precipitation in a solution for methyl parathion electrochemical detection [39]. The hybrid diatom-based electrode outperformed in the limit of detection (at picomolar level) when compared with many other electrodes (only active at nanomolar level), even if all modified with other nanostructured materials.

## 3. Diatoms as SERS Substrates

**Figure 3:**
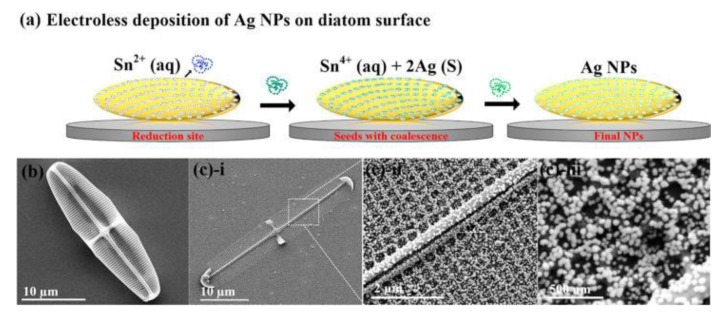
Schematic representation of Ag NPs’ growth on a diatom surface (**a**) FE-SEM top-view images of diatom (**b**) and in situ synthesized Ag NPs on diatom surface; (**c**) i–iii various magnifications of the NP-populated diatom. Reprinted with permission from [40]. Copyright 2019 American Chemical Society.

Surface-enhanced Raman scattering (SERS) has gained increasing attention by research groups for sensing and identification of substances in trace. By analyzing the vibrational absorption spectrum, it is possible to label-free detect chemical and biological complexes with very high sensitivity and specificity. The fabrication of plasmonic active SERS supports requires the top available nanofabrication techniques, which are not widespread in all research laboratories. The group of Prof. G. Rorrer at Georgia State University, USA, firstly used metal nanoparticles modified diatoms frustules as highly performing SERS substrates [40]. The group continued to publish new articles on this topic. The authors applied this technology to the detection and identification of explosive molecules in nanoliter solutions, such as melamine at very low concentration (1 ppm) and also Tetrahydrocannabinol (THC) in body fluids [41,42,43]. These works demonstrated that the diatom frustules could be considered bio-derived, cost-effective, modified nanostructured supports for SERS experiments with significant potential in applications ranging from healthcare to food testing and environmental monitoring. The best results have been obtained by diatoms modified by silver (Ag) nanoparticles (NPs) directly growth on the biosilica surface by electroless deposition, as can be seen in Figure 3. The fabrication method assured a high density of Ag NPs on the diatom surface, which meant a large amount of plasmonic hot spots that enhanced the Raman signal. The enhancement factor allowed a detection limit of THC in methanol down to 10^−12^ M that should be compared with standard SERS substrate (i.e., Ag NPs on a glass slide) performance of only 10^−7^ M.

Gold (Au) NPs modified diatom biosilica has been used by Kaminska et al. as ultrasensitive SERS substrates for the detection of interleukins in blood plasma [44]. The use of diatom frustules enhanced the sensitivity of the technique down to picograms per mL of interleukin 8, whereas other substrates allowed the detection of only nanograms per mL.

Wang et al. used the frustules obtained by diatomite, which is fossilized remains of ancient diatoms as geological deposits, currently used in industry as water filters, adsorbents, and also in toothpaste and food integrators [45,46]. The diatomite frustules, integrated into a proper microfluidic circuit, could quantify illicit drugs, such as pyrene and cocaine, at ppb level in the human plasma.

## 4. Diatom-Based Biosensors

The photoluminescence features of diatom frustules are still under discussion [47]. Three main emission contributions are classified. The first one is related to the excitation and emission in the ultraviolet region between 250 and 300 nm. This activity is normally attributed to the organic fraction entrapped in the biosilica structure. The second type is associated with the emission in the blue region of the visible spectrum, and the third type of photoluminescence activity is characterized by strong emission in the green at wavelength 523 nm. The origin of visible emission in the blue and green regions is due to the different defect states, such as oxygen defect centers as non-bridging oxygen hole centers or neutral oxygen vacancy and self-trapped excitons. By using the photoluminescence modulation in antibody functionalized diatom frustules of *Amphora* sp., Viji et al. detected BSA protein at mM level, while Selvaraj et al., using the same species, selectively detected *Salmonella typhi* with a detection limit of 10 pg [48,49].

**Figure 4:**
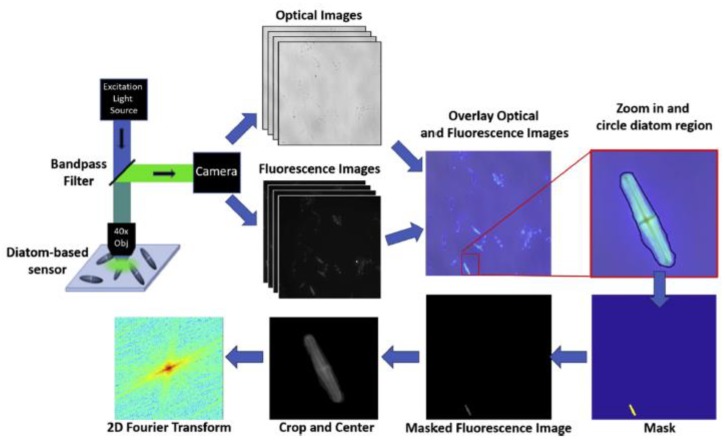
Schematic of quantification of the signal from imaged diatom frustules. Reprinted with permission from [52]. Copyright 2019 Elsevier.

The hierarchical, quasi ordered disposition of porous nanostructures in diatom frustules has been exploited by the Rorrer group for demonstrating a photonic crystal enhanced fluorescence imaging immunoassay able to detect target analytes for cardiovascular disease and IgG molecules [50,51]. The pores of the diatom *Pinnularia* sp. frustules used were under 100 nm in size, arranged in such a way that the optical fields were enhanced by the Purcell effect. As can be noted in Figure 4, the functionalized frustules could be simply imaged by fluorescence microscopy and the image rapidly analyzed to get quantitative information. The coupling between the fluorophore emission and the optical field form the diatom surface allowed a limit of detection of the mouse IgG down to 10^−16^ M (14 fg/mL) and of a few pg/mL of the hormone N-terminal pro-B-type natriuretic peptide. These results were 100× and 2× better than others in the literature, respectively.

Lim et al. used fluorescence molecularly imprinted polymer based on *Navicula* sp. frustules for lysozyme sensing. The authors demonstrated a limit of detection equal to 1.5 µg/mL [52].

## 5. Conclusions and Perspectives

Diatoms are present in marine and freshwater all over the world; moreover, diatoms can be easily cultivated on a very large scale in simple reactors and mild conditions. These characteristics assure a good availability at a low cost of this potentially technological material. Scientists and technicians working on the transducers for biosensing applications often face the compromise between their costs and performances. This is particularly true in the case of nanostructured materials that could require sophisticated fabrication processes. In the last few years, biomimetic, i.e., the emulation of nature for inspiring new solutions to technological problems, is an alternative approach to innovation. Diatoms are natural models of complex architectures with unbelievable consequences in optics and also in mechanics and biomedicine not yet fully explored by the scientific community [53]. The diatom-based biosensors recently reported in literature exploit the photoluminescent emission of the frustules or the porous ordered morphology of their surface to obtain extremely good SERS substrates. Other research groups are investigating the optical transmission features of diatom frustules that, beside the relevant biological implications, could be used in the next future for biosensing purposes [54,55,56]. The quality of the works recently published is the best pointer for a new route toward an exciting field of scientific and technological exploration. Material scientists and all the people involved in specific applications of multifunctional supports will strongly benefit from the wonderful world of diatoms.
